# Agent-Centred Nuclear Safety Principles for Small Modular Reactors

**DOI:** 10.1007/s11948-026-00629-5

**Published:** 2026-09-09

**Authors:** Johanes Narasetu Widyatmanto, Rafaela Hillerbrand

**Affiliations:** Philosophy of Engineering, Technology Assessment and Science (PhilETAS) Research Group Institute of Technology Assessment and Systems Analysis (ITAS), Karlsruhe Institute of Technology, Karlsruhe, Germany

**Keywords:** Small modular reactors, Nuclear safety principles, Ethical risk analysis, Responsibility distribution, Agent-centred safety principles

## Abstract

Small Modular Reactors (SMRs) are among the new designs that promise more compact nuclear power plants (NPPs) with scalable generation capacity and shorter construction time, while retaining emission-free electricity in exchange for small amounts of manageable radioactive waste per kWh of generated electricity. Their size and modular design contribute to the growing popularity of SMRs. However, these features also present risk scenarios that do not apply to large, conventional NPPs, such as when a buyer and SMR manufacturer are located in different nations with different regulations and standardisations. Such potential new scenarios require explicit ethical considerations. Unfortunately, such considerations are absent from the International Atomic Energy Agency (IAEA) safety principles, particularly for SMRs. Existing nuclear safety principles are too broad to keep up with potential new scenarios introduced by the novel features of SMRs. Meanwhile, existing approaches in nuclear energy ethics are focused on specific nuclear-related issues across reactors, such as radioactive waste management and nuclear fuel processing. Drawing on Hermansson and Hansson’s agent-centred ethical Risk Assessment (eRA), this paper aims to enhance the IAEA’s nuclear safety principles in order to clarify the distribution of responsibilities for nuclear actors, especially in SMR cases. We articulate what we call the agent-centred presupposition in eRA and apply it to nuclear safety principles using the case of SMRs. We then propose a critique of this approach and suggest further conceptual analysis, which could be suitable for considering SMRs or new reactor designs more generally.

## Introduction: The Challenges of SMRs for Principles-Based Approaches to Nuclear Safety

Small Modular Reactors (SMRs) are one of several evolutionary and innovative designs (EIDs) for nuclear power plants (NPPs) being promoted by the International Atomic Energy Agency (*Applicability of Design Safety Requirements to Small Modular Reactor Technologies Intended for Near Term Deployment, *2020). As the name suggests, these reactors have a smaller generation capacity and more compact design than traditional NPPs. They are scalable and consist of modules that prevent complete reactor shutdown, thus promising a stable electricity supply even during maintenance. These features allow SMRs to be used in smaller spaces and sited near the area of usage, such as housing, e-fuels production, electrolysers, and even AI data centres (Moore, 2024, World Nucl. News 2024a; Eccles, 2024). In light of such broad applications, nuclear companies and research centres have developed various SMR designs. To date, there are 98 SMR designs around the world in various stages of development, of which five are under construction and two are already in operation (Nucl. Energy Agency NEA 2024).

Along with the promise SMRs hold for the nuclear industry, some of their novel features pose new scenarios that do not apply to large reactors. For example, their modularity and compactness allow reactors to be constructed in one country then shipped to another, with each state potentially having different experiences with nuclear power and different safety regulations. With more SMR designs being approved, entering the construction stage, and bringing new multi-state implementation scenarios, it is essential to have an ethical framework that anticipates these designs. Such a framework must provide clear and comprehensive guiding principles for SMR engineers, business actors, and interested policymakers to safely design and operate these reactors in the market.

From the perspective of international policy, the International Atomic Energy Agency (IAEA) has published guidance specific to SMRs, with ‘safety first’ as its highest principle (see, e.g., *Experience in Applying IAEA Principles for Design Safety to New Nuclear Power Plants,*^2023^; Dusic et al., 2014; *Applicability of Design Safety Requirements to Small Modular Reactor Technologies Intended for Near Term Deployment,*^2020^). The new features introduced with SMRs must comply with nuclear safety standards, which the IAEA has defined as “the achievement of proper operating conditions, prevention of accidents and mitigation of accident consequences, resulting in protection of workers, the public and the environment from undue radiation risks” (*IAEA Nuclear Safety and Security Glossary,*^2022^). This definition of nuclear safety seems to align with the IAEA’s ten safety principles, which guide nuclear practice around the world (Table 1, Appendix).^1^ These principles lay out what needs to be done on the technical, managerial, and regulatory levels as well as by the responsible actors in order to achieve nuclear safety when building nuclear facilities. However, because of the principles’ broad applicability to all types of nuclear facilities – for electricity production, medical purposes, food safety, etc. – further specification is needed to address the safety-related challenges posed by new reactor designs, particularly SMRs.

**Table 1:** The IAEA fundamental safety principles (Fundamental Safety Principles, 2006)

Fundamental Safety Principles	Definition
1. Responsibility for safety	The prime responsibility for safety must rest with the person or organisation responsible for facilities and activities that give rise to radiation risks.
2. Role of government	An effective legal and governmental framework for safety, including an independent regulatory body, must be established and sustained.
3. Leadership and management for safety	Effective leadership and management for safety must be established and sustained in organisations concerned with, and facilities and activities that give rise to, radiation risks.
4. Justification of facilities and activities	Facilities and activities that give rise to radiation risks must yield an overall benefit.
5. Optimisation of protection	Protection must be optimised to provide the highest level of safety that can reasonably be achieved.
6. Limitation of risks to individuals	Measures for controlling radiation risks must ensure that no individual bears an unacceptable risk of harm.
7. Protection of present and future generations	People and the environment, present and future, must be protected against radiation risks.
8. Prevention of accidents	All practical efforts must be made to prevent and mitigate nuclear or radiation accidents.
9. Emergency preparedness and response	Arrangements must be made for emergency preparedness and response for nuclear or radiation incidents.
10. Protective actions to reduce existing or unregulated radiation risks	Protective actions to reduce existing or unregulated radiation risks must be justified and optimised.

As for philosophical approaches to nuclear safety, existing accounts focus on the back-end process of nuclear technology writ large, such as high-level radioactive waste (HLW) management or the engineering process and policy practice for conventional, large reactors.^2^ However, radioactive waste management addresses just one of the ten safety principles. In view of this, a philosophical approach that accounts for all ten safety principles and addresses the new risk scenarios made possible by SMR designs is needed.

Countries or industries considering the adoption of nuclear energy will need to assess various reactor designs, and a clear safety guide could favour some designs over others. One safety consideration that could favour conventional large reactors is simplicity of control. Because large reactors are built on-site, safety is easier to control, while reactor modules for SMRs are assembled in a factory, potentially making safety assurance more difficult. We do not dismiss other considerations, including sustainability (Taebi & Kloosterman, 2015), democratisation of nuclear technology (Pols & Spahn, 2015), context-related societal values (Grunwald, 2015), a culture of responsible nuclear engineering (Franssen, 2015), or future societal outlook (Zwart, 2015). However, because safety is often regarded as the highest priority to consider when building NPPs, the currently undertheorized safety guides applicable to SMRs from the IAEA and philosophers motivate us to develop nuclear safety principles which are relevant for SMRs in particular, and for future innovative reactor designs more broadly.

In order to develop SMR-relevant nuclear safety principles, we apply the concept of agent-centred risk distribution underlying Hermansson and Hansson’s ethical Risk Assessment (eRA) to the IAEA’s ten fundamental safety principles. Section “The advantages and ethical challenges of SMRs” discusses features of SMRs and the ethical challenges they pose. Section “Ethical Risk Assessment (eRA)” explains eRA and its relevance to SMRs. Section “How could the eRA’s” applies this underlying concept to reformulate the IAEA’s fundamental nuclear safety principles. Section “Critique: eRA’s Agent-Based Approach Overlooks Institutional Embedding as individuals’ Milieu” reviews the shortcomings of our revised nuclear safety principles and what could be addressed in future analyses. Section “Conclusion” concludes the paper.

## The Advantages and Ethical Challenges of SMRs

SMRs are promoted as solutions for energy security in transitioning away from fossil fuels due to the key features they share across various designs, including modularity and compactness. In addition, some studies indicate problems specific to NPP construction that could be addressed by SMRs, such as cheaper construction costs and faster construction time. If their potential is realised, these reactors could satisfy the need for a reliable technology for the energy transition, delivering abundant and stable electricity in a short amount of time while keeping costs low. However, the ethical issues often discussed in connection with the civil use of nuclear power plants also apply to SMRs, with additional use cases that are specific to small reactors. Therefore, while the general ethical approaches associated with nuclear energy are still applicable, the concrete scenarios of SMRs require a novel application of these approaches. In this section, we explain four key potential advantages of SMRs, followed by the ethical challenges they pose.

The following are often presented as promising features of SMRs:

- *Modularity and scalability.* In an SMR, the whole reactor unit is one module constructed in a factory and transported to the site. Compared to large reactors, where all construction takes place on-site in an integrated NPP system custom-made for that location, SMRs allow for factory standardisation (Pannier & Skoda, 2014). As a result of this modularity, SMRs are also scalable, such that their small generation capacity could more easily adapt to a gradual increase in demand by adding more reactors to the same site.
- *Compactness in the smaller reactors’ capacity.* Smaller generation capacity also leads to a more compact design, allowing SMRs to be dispatched to more diverse locations such as isolated towns. For instance, Russia’s operational SMRs with their floating design supply heat and electricity to the town of Pevek and are intended to power colder and more remote Arctic communities (*Small Modular Reactors: Advances in SMR Developments 2024,*^2024^).
- *Faster construction time.* Because the reactor module is physically smaller, the required construction time for an NPP unit of SMRs could be reduced. An NPP consisting of several SMRs normally requires around three to five years to build, which is significantly faster than a conventional NPP’s construction time of seven to ten years (Pannier & Skoda, 2014).
- *Lower upfront cost compared to large NPPs.* Due to SMRs’ smaller physical size, lower generation capacity, and faster construction time, this new design could potentially cost less than conventional large NPPs. In the United States, a conventional NPP costs approximately 14 to 30 billion USD (Lovering et al., 2016) versus an estimate from NuScale, an American SMR company, which gives an approximate cost of 5.3 to 9.3 billion USD. However, the estimated construction cost depends on many factors. The longer a project is delayed, the more dramatically cost estimates could rise (Schlissel, 2023).

To date, there are two operational commercial SMRs, one in China and another in Russia. Chinese SMRs were built by China Huaneng Group Co. to supply electricity for Shandong province (*Bloomberg.Com,*^2021^; Song, 2021). Meanwhile, in Russia, a state-owned company named Rosatom has commercial SMRs located in Yakutia (World Nucl. News, 2024b; Kuznetsov, 2021). While global-scale SMR commercialisation still seems to be a distant possibility, it is safe to say that there is already a great deal of optimism about this new scale of reactor technology.

While SMRs offer important advantages in the smaller size and modularization of nuclear reactors, they also pose significant ethical challenges. We consider the following four scenarios to hold the greatest ethical relevance for SMRs:

- *Fairness to the public: higher chance of privatisation for industrial use.* Some industries, such as AI data centres, require enormous amounts of electricity to operate continuously (Cha, 2025). On the one hand, having a separate industrial grid reduces the burden on other users, such as cities and domestic consumers. Moreover, large investment funds could make the argument that SMRs belong entirely to whoever pays for them. However, privatisation of electric grids for industrial purposes raises the question of the extent to which transparency and responsibility is needed from the industry actors. A clear agreement between relevant actors on rights, responsibilities, and data transparency and accessibility could be helpful here.
- *Safety: potential for reactor accidents to impact a larger number of people due to proximity to cities.* Making reactors smaller and transportable opens up the possibility of installing SMRs closer to cities (An et al., 2025). An argument could be made for a greater safety risk associated with reactor accidents in a populated area. This raises the question: to what extent is it acceptable to site nuclear reactors close to populated areas? Continuous public outreach fostering public discourse on the safety of the reactor project should thus be promoted.
- *Security: interference during transport could entail losing an entire reactor module.* In contrast to large NPPs, where reactor assembly takes place on-site, SMR reactors are constructed as modules in a factory. This module is then transported to the construction site by sea or land (Lloyd et al., 2021). There is a security-related argument to be made regarding access to an entire reactor assembly by malicious actors during transport, raising the question of who should be responsible for what, as well as the extent of that responsibility. Adequate security during the transportation process and limited accessibility to the modules should thus be established.
- *Fairness to embarking countries: keeping SMRs’ engineering knowledge in the countries of production.* Because an SMR reactor is a module constructed in a factory for shipment to the buyers, issues of knowledge ownership might also arise. We can ask whether it is fair to restrict the knowledge of SMR production to the countries that sell them, thus creating dependencies for the countries that want to use nuclear power for their grid, but cannot produce it themselves. This is not to say that SMR producers should have no rights over their inventions. However, when clean, safe, and non-intermittent energy could provide electricity to many people, particularly those in energy poverty, it would be fairer to gradually transfer the engineering knowledge so that the countries purchasing SMRs would eventually be less dependent on the producing countries.

These challenges revolve around the new scenarios of reactor construction, transport, and operation that arise from the smaller size and modular nature of the reactors. While some of these difficulties are also applicable to large reactors, SMRs accentuate the need to revisit existing NPP issues in light of certain novel challenges related to smaller reactors.

These challenges could be better addressed with clear ethical guidance throughout the entire process of designing, engineering, operating, and decommissioning SMRs. However, existing IAEA nuclear safety principles (Table 1 in the Appendix) are not specific enough to address the issues of future reactor designs presented by SMRs. We therefore consider current nuclear safety principles insufficient for guiding action in the case of incidents.

To better guide nuclear engineers, industry actors, and policymakers navigating the dynamics of commercialising SMRs, we propose to revisit existing nuclear safety principles, making them more specific so as to address SMRs and other potential advanced reactor designs (such as micro-reactors) that might come in the future.

We draw on the framework of Hermansson and Hansson’s ethical risk assessment (eRA) for two reasons: its explicit emphasis on ethics in assessing risks, and its purpose, which is to supplement existing techno-economic risk-based approaches. This approach complements existing accounts on both nuclear safety in general and specific issues that arise with EIDs such as SMRs. eRA’s focus on the individuals involved in risk scenarios allows it to address what risk assessments often leave out, namely, the insufficient characterisation of consequences, moral issues, and future perspectives on today’s decisions (Hansson, 2016).

## Ethical Risk Assessment (eRA)

### The Agent-Centred Premise of eRA

**Fig. 1:**
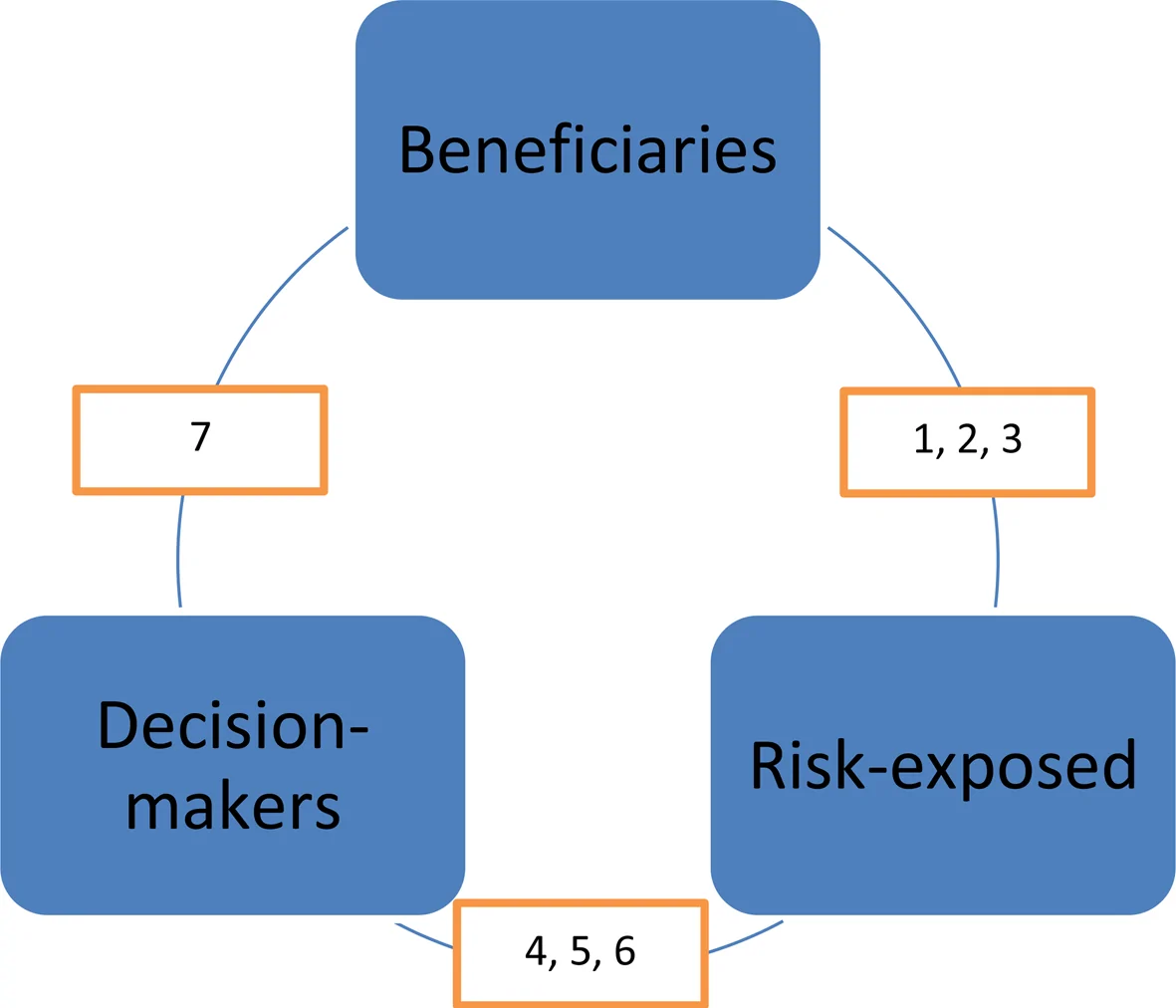
Hermansson and Hansson’s three-party model for ethical risk analysis [8]0. 1. To what extent do the risk-exposed benefit from the risk exposure (Q1)? 2. Is the distribution of risks and benefits fair (Q2)? 3. Can the distribution of risks and benefits be made less unfair by redistribution or compensation (Q3)? 4. To what extent is the risk exposure decided by those who run the risk (Q4)? 5. Do the risk-exposed have access to all relevant information about the risk (Q5)? 6. Are there risk-exposed persons who cannot be informed or included in the decision process (Q6)? 7. Does the decision-maker benefit from other people’s risk exposure (Q7)?

Hermansson and Hansson’s three-party model of an ethical risk analysis (Fig. 1, Appendix) examines the relationship between the risk-exposed, the beneficiary, and the decision-maker in risk distribution (Hermansson & Hansson, 2007, 2018). This three-party model analyses risk and responsibility distribution and tackles the problem of great uncertainty or ignorance by asking seven moral questions to elucidate how each party is exposed to risk and the extent of their responsibilities for such risk. According to Hermansson and Hansson, the seven moral questions proposed here are open to modifications, and what constitutes fairness in risk distribution also depends on our choice of moral account. These points allow some flexibility in applying eRA, so that it can potentially be applied as an assessment based on the principle of distributive justice (Hansson, 2017c).

Note that distributive justice requires further conceptual clarity. If SMRs allow industries and families to fulfil their needs, nuclear risks threaten this. We recommend basic capabilities as parameters for fair risk distributions because this approach addresses the ethical dimension of (radioactive) risk at a foundational level: the threat to exercising basic capabilities, hence possibly limiting or eliminating the possibility of human flourishing. On this topic, Sen criticises a resource-centred approach for not asking what these resources do, given their distributions across different socio-cultural contexts (Sen, 1987). For Sen, a fair distribution of advantages or disadvantages is judged in terms of how it promotes or reduces combinations of human capabilities (Sen, 2011) by providing sufficient space for individuals to function (Sen, 1992, 1993). In the distribution of radioactive risk, viewing fairness in terms of capabilities encourages us to minimise risks instead of simply assigning them to different parties.

Hermansson and Hansson note that ethical questions in eRA are open to addition or correction, because the eRA is intended to engage in risk analysis with not only quantitative measures, but also qualitative-normative questions (Hermansson & Hansson, 2003, 2007, 2018). With regard to roles in risk-benefit distribution, Hansson describes how individuals in their different roles – as risk-exposed, decision-makers, or beneficiaries – are prone to multiple exposures to risks and benefits (Hansson, 2017a). Furthermore, per Hansson, the division of roles is not exclusive, so a person could be both a decision-maker and a beneficiary, a beneficiary and risk-exposed, a decision-maker and risk-exposed, or all three at the same time (Hansson, 2018).

According to Hermansson and Hansson, identifying ethical aspects of a risk problem should be as much a matter of course as quantifying probabilities and consequences in risk analysis (Hermansson & Hansson, 2007). To enable this, the distribution of risks and benefits needs to be clearly mapped. In this regard, the three-party model of an ethical risk analysis does not replace various established probabilistic risk assessments (PRAs). Rather, it proposes to integrate two important elements into PRAs. First, the model proposes a map of those who impose and benefit from the risk at hand. Second, the model suggests seven additional ethical questions to incorporate into existing risk assessments. With the focus on distributive justice^3^ in these seven ethical questions, eRA is distinguished from and complements cost-benefit analysis, which has a tendency to omit an analysis of distributive effects.

Figure 1 in the Appendix shows Hermansson and Hansson’s three-party model of an ethical risk analysis along with the seven reflective questions (Hermansson & Hansson, 2007, 2018). Each of the seven questions considers aspects which cannot be described by or are difficult to address in terms of probabilities. In general, these ethical questions are agent-based due to their focus on qualitative human relations in connection with risks and benefits. These agent-centred questions identify who is at risk and how, who benefits when the risks are imposed and how, and what role each agent plays in relation to the distributed risks-benefits:

- *Q1. To what extent do the risk-exposed benefit from the risk exposure?* While some parts of this question can be addressed by quantitative economic analysis, not all benefits and risks can be translated into monetary value. By asking about the extent of benefits instead of a more specific economic account, this question acknowledges that not all risks and benefits for an agent are quantifiable, let alone monetizable.
- *Q2. Is the distribution of risks and benefits fair?* This question aims to ensure that those who are already exposed to risks will not be further exposed to additional risks. The question of fairness in risk-benefit distribution addresses the issue of ignorance. Rather than accounting for the probability of risks, this question considers how risks, independent of our ability to estimate them, are distributed among the parties involved, and whether or not such distribution is acceptable *vis-à-vis* the likely benefits.
- *Q3. Can the distribution of risks and benefits be made less unfair by redistribution or compensation?* Compensation for accepting risk could be perceived as some sort of bribery, as mentioned by Thomas, among others (Thomas, 2023). The alternative to this is to offer an exchange not in the form of money, but of something ‘in-kind’. The question of compensation can of course easily be interpreted as a matter of calculation if the risk at hand can be translated into monetary terms and the compensation is an object with equal monetary value. However, one could also go beyond monetary calculation by considering compensation in terms of social benefits (see Van Est et al., 2023). The agent-based nature of this question lies in its emphasis on the social aspects of a fair distribution of benefits in addition to economic factors.
- *Q4. To what extent is the risk exposure decided by those who run the risk?* This question aims at mapping the relation between decision-makers and the risk-exposed with regard to the specific risks under assessment. The relation between parties is not dichotomous, where decision-makers are fully responsible for imposing risk on the risk-exposed. Rather, the risk-exposed could also contribute to the risk through their own decisions, such as when a local community in a democratic system chooses to commit to one type of power plant over another. The agent-based nature of this question promotes public discourse about the risks and benefits of a technology, so that beneficiaries and decision-makers can acquire a more holistic understanding of the significance of a risk-inducing project.
- *Q5. Do the risk-exposed have access to all relevant information about the risk?* This question highlights the accessibility of relevant information for the risk-exposed. Risk information is understood as relevant for the risk-exposed if knowing it would affect a person’s actions and decisions with regard to risk. Taking a broad understanding here, risk information is considered to be relevant for the risk-exposed if knowing it would affect a person’s actions and decisions with regard to risk. This question highlights the importance of developing agents’ knowledge about new technologies, since, as new technological development arises, important new information could emerge.
- *Q6. Are there risk-exposed persons who cannot be informed or included in the decision process?* This question can be interpreted as seeking to include as many risk-exposed people as possible in the decision process. However, it can also be understood as calling attention to the fact that some risk-exposed people cannot be included in this process, such as future generations. By attempting to include future generations about whom we have no information, the question could also be seen as addressing the issue of ignorance, since it seeks to include the unknown perspectives and/or interests of those who do not yet exist.
- *Q7. Does the decision-maker benefit from other people’s risk exposure?* This question aims to assess the trustworthiness of decision-makers in connection with the risks resulting from their decisions. If they benefit from other people’s risk exposure, they might lose credibility, and the general public might not cooperate with future policies. Unquantifiable benefits such as reputation and trustworthiness are also agent-centred aspects.

Instead of seeking to estimate probabilities, this approach involves questions about the extent of risk exposure (Q1), the fairness of risk exposure (Q2–Q4), the state of being risk-informed (Q5–Q6), and the benefit from risk exposure (Q7). These moral questions highlight the centrality of agents with a varied mix of roles in a risk scenario, and this agent-centredness can be implemented within nuclear safety principles when assessing concrete SMR issues.

### Relevance of eRA to SMRs

These seven questions address the issue of fair distribution of risks and benefits in large-scale activities. Turning our attention to SMRs, we consider two specific real-world scenarios to be noteworthy: the adoption of SMRs as the first-of-a-kind (FOAK) nuclear power plant by countries with no previous experience handling NPPs; and complete grid privatisation made possible by smaller, modular reactor designs.

*Scenario 1. Building SMRs as the first-of-a-kind (FOAK) NPP in inexperienced countries*. Like large NPPs, SMRs come with various designs, the majority of which are experimental. It is ethically problematic when an untested SMR design is endorsed for countries that are entirely new to nuclear energy, with no previous experience even with established commercial large NPPs. For instance, Indonesia has no experience in commercial NPPs and is considering building SMRs. One of the options is a molten-salt SMR from a Singapore-based company, which has never been attempted before (World Nucl. News 2025). Here, it is important to reflect on the fair procedure for commercialising experimental reactors in inexperienced countries. In particular, we can examine the accessibility of the SMRs’ technical design, financing, emergency scenarios, and the decision-making process in order to ascertain the fairness of putting unpredictable technologies to the test in inexperienced countries, and consider more responsible ways to build NPPs in these countries.

*Scenario 2. Buying SMRs as a private grid to power large industries.* SMRs’ modularity and compactness make the technology technically feasible for ownership by private entities such as AI data centres. Cha (2025) has argued that the rising use of energy by AI data centres requires a compact power source that is able to both provide enough energy and be scaled to anticipate future increases in demand. Privatisation of power plants is not new; the backup generators that industries have long possessed to sustain their activities could be viewed as private power plants. However, ethical principles for the responsible use and management of a private nuclear-powered grid are rarely addressed, and a closer examination of national legislations is necessary to ensure that they protect against high-impact risk from private grids. In order to ensure such responsible management, principles of fair risk-benefit distribution must be established.

With the above real-world scenarios, eRA is relevant for assessing the risks of SMRs and, by extension, other future reactors with various designs, sizes, and generation capacities. This relevance is justified for two reasons. First, given SMR characteristics (modularity and compactness), their novelty (many designs are untested), and the different experience level of their users (inexperienced countries or companies), the risk-benefit distribution of applying these technologies is less predictable than the existing use cases of large nuclear reactors. eRA takes these uncertainties into account and offers agent-centred ethical reflections that do not require a strict quantification of risk-benefit distribution. Instead of quantifying risks, eRA focuses on how and to what extent different agents with different roles in a given scenario are at risk and/or might benefit from the scenario. From there, one can reflect on whether the existing risk-benefit distribution in a given scenario is ethically acceptable, and then look for a responsible way to manage risky activities by further applying this ethical framework to safety principles for nuclear power plants.

Second, it is reasonable to apply eRA to SMRs and other potential new NPPs due to the compatibility of eRA’s ethical framework with nuclear safety principles. The IAEA’s nuclear safety principles propose measures to minimise radioactive risks. Meanwhile, eRA invites us to consider that risk scenarios include multiple agents who benefit differently with regard to risk distribution. Applying eRA to nuclear safety principles allows us to analyse the ethical acceptability of a risk-benefit distribution scenario by reflecting on who should be responsible for applying measures to minimise these risks. In the two scenarios mentioned above, inexperienced countries and private industries prefer SMRs because of the scale of their electricity needs, which small reactors happen to cater to best. Power plants with small size, high scalability, and abundant, stable production gain priority *despite* inexperience with nuclear power and uncertain risk-benefit distribution, with potential implications for fairness issues. The eRA framework suggests that, even in uncertain, risky scenarios, agents are responsible for creating a fair risk-benefit distribution.

Examining the different roles of the agents in a responsible risk-benefit distribution is essential for the ethical use of nuclear power, including the introduction of reactor technologies with significant uncertainties, as exemplified by SMRs. In relation to the IAEA international legal framework with ‘safety first’ as its core value, it is ethically critical to translate safety into actionable principles that can anticipate new designs. Unfortunately, the existing fundamental safety principles do not specify the different roles of agents in a nuclear scenario or how those agents are responsible for a fair risk-benefit distribution. The next section will articulate what fundamental nuclear safety principles would look like when fairness in risk-benefit distribution among the various agents is considered.

## How Could the eRA’s Agent-Centred Perspective Be Applied to Nuclear Safety Principles?

eRA complements the quantitative approach to risks because it does not aim to assign probability estimates, nor does it suggest monetary risk compensation measures. Instead, the questions posed in eRA emphasise relations between agents in a risk scenario and address the problem of fairness by examining how risks and benefits in such a scenario are distributed. This section considers how eRA’s agent-based risk-benefit distribution could enhance the IAEA’s fundamental safety principles (Fundamental Safety Principles, 2006) such that they are more applicable to EIDs such as SMRs. These safety principles are *prima facie* ethical principles that implement the uncompromisable value of safety in building NPPs while still allowing companies and regulatory bodies to interpret them in engineering and business practice. We will proceed by outlining the IAEA nuclear safety principles and offering their agent-centred reformulation (this comparison is summarised in Table 2).

- *Principle 1: Responsibility for safety*

The IAEA states that the prime responsibility for safety must rest with the organisations or persons responsible for activities which create radioactivity. In connection with ‘who is responsible for what’, eRA’s contribution lies in mapping the parties involved in the rise of radioactive risk. Such mapping shows that while beneficiaries, decision-makers, and the risk-exposed (such as workers in an NPP) contribute to radioactive risk in different ways, each party can be said to enable activities which increase radioactive risk. Even so, decision-makers and beneficiaries should bear more responsibility than the risk-exposed. If, according to the IAEA, the responsible parties are those whose activities and/or facilities increase radioactive risk, then we can articulate principle 1 from an agent-centred perspective as follows: the prime responsibility for safety must rest with the persons or organisations *that benefit from and decide to initiate* facilities and activities that give rise to radiation risks.

Applying the newly formulated principle 1 to the SMR context, we can be more specific in distributing responsibility primarily between policymakers and SMR companies. For example, in the development of molten-salt SMRs in Indonesia, where the decision to introduce SMRs took place mainly through discussions between policymakers and SMR manufacturers, one can ask whether most responsibility should be borne by the SMR company (ThorCon, or other companies in the future), the local government, or the Indonesian National Energy Assembly. This reflection ensures a clear course of action to be taken by the respective parties in case of safety accidents.

b. *Principle 2: Role of government*

The IAEA states that there must be an effective legal and governmental framework for safety with an independent regulatory body. The government and regulatory bodies are tasked with establishing standards and regulatory frameworks to protect people and the environment from radiation risks, with the primary responsibility held by the licensee. From eRA’s agent-centred perspective, principle 2 could be formulated thus: there must be an effective legal and governmental framework for safety with an independent regulatory body *assessing radioactive risk exposure, benefits derived from radioactive risk, and the distribution of both*.

In the SMR case, agent-centred principle 2 can be applied by looking at the relation between the government and the SMR company. For instance, in countries like Indonesia, which are not just new to SMRs but to NPPs, the radioactive risk could be higher due to unknown factors, such as preparing local engineers to operate and maintain this new technology for generations to come. With this higher radioactive risk, we need to ensure that both the government and SMR companies constantly involve local engineers and energy research centres as a form of responsibility in building sustainable SMRs. This can be done by mapping the potential area of radioactive risk should accidents happen, listing the benefits of SMRs for different groups, such as secured investment, stable electricity for domestic, agricultural, or industrial use, etc. Regulators could then formulate laws and directives to ensure radioactive risk is kept at a minimum while the benefits for society remain significant.

c. *Principle 3: Leadership and management for safety*

This principle is concerned with establishing and sustaining leadership and management for safety, not only in facilities and activities that give rise to radioactive risks, but also in other organisations that are connected with these risks. The leadership and management of nuclear facilities stipulated in this principle could benefit from eRA’s questions about the risk-exposed. Power plant workers are also exposed to radioactive risk as they build and maintain NPPs. With moral questions from eRA about knowledge accessibility for working engineers as the risk-exposed within nuclear facilities, agent-centred principle 3 could be formulated thus: it is compulsory to establish and sustain leadership and management for safety in nuclear facilities that give rise to radioactive risks *by, among other things, ensuring the accessibility and distribution of knowledge about radioactive risks to all relevant agents*.

This agent-centred principle 3 is applicable to new reactors such as SMRs. Innovative features such as modularity, smaller and more compact reactor design, and the choice of nuclear fuel cycle should be understood by working engineers from SMR companies as well as governmental and non-governmental experts assessing the feasibility of SMRs.

d. *Principle 4: Justification of facilities and activities*

With this fourth principle, the IAEA seems to use a risk-benefit approach as it states that “facilities and activities which give rise to radiation risks must yield an overall benefit”. The “overall benefit” here is measured by the consequences of nuclear facilities.^4^ In this regard, eRA could contribute by specifying what constitutes a benefit, for whom, and whether or not such benefits at the expense of radioactive risks could be considered fair. In terms of basic capabilities, the fairness of the distribution and exchange of risks and benefits is measured in a sufficientarian way, i.e., by analysing how such distribution and exchange provide an adequate space for human flourishing in one’s concrete socio-cultural context. Additionally, eRA also allows for compensation, either monetary or in-kind. With an agent-centred perspective, principle 4 could be formulated as follows: facilities and activities which give rise to radiation risks must yield an overall benefit *either monetarily or in-kind for the risk-exposed located near the nuclear facilities.*

With the example of nuclear reactors such as SMRs, agent-centred principle 4 could be applied by assessing whether there are any benefits beyond monetary concerns when building SMRs. Countries like Indonesia, where the energy grid is dominated by fossil fuels, particularly lignite, with the advantage of cheap electricity, could reflect on whether applying SMRs would not only maintain cheap electricity, but also provide a more stable, emission-free supply. In this case, what constitutes a benefit could include environmental and health considerations in addition to economic factors.

e. *Principle 5: Optimisation of protection*

The IAEA states that it is necessary to optimise protection “to provide the highest level of safety that can reasonably be achieved”. This principle does not further specify the meaning of ‘reasonable’. It could be interpreted as an implementation of all currently available knowledge about nuclear energy so as to engineer facilities with next-to-no radioactive risks. That said, more could be added to better articulate the idea of providing the highest possible level of safety.

In the context of NPPs, there is also a risk of reducing reasonability to economic viability. This consideration leads certain EIDs, such as fusion reactors, to remain out of commercial use due to the cost of the technologies. However, limiting reasonability to economic viability could compromise optimisation of protection. The agent-centred perspective could contribute by specifying elements of fair risk distribution by the decision-makers and nuclear companies as the beneficiaries in order to optimise protection. The agent-centred principle 5 could thus be: *governments and nuclear companies* must provide the highest possible level of safety *with a reasonable standard that adheres to fair radioactive risk distribution*.

For example, in building SMRs in Indonesia, the local and national government must first establish regulations for nuclear energy, which are also applicable to new reactors, before construction begins. This regulation is also based on a continuous feasibility study involving not only the company’s engineers, but also research centres. In the field, NPP companies must ensure that their licensed SMR design is precisely constructed in terms of modularity, passive safety systems, the choice of thorium as both coolant and moderator for the reactors, adaptive construction to seismic activities, and so on. These aspects ensure the safety of the SMR workers as well as the people living in the vicinity of SMRs who draw electricity to carry out their daily activities. In the case of SMR for industrial use, it also ensures the industry maintains its activity. Fair risk distribution comes in minimising radioactive risk for the people in the vicinity while enhancing their capabilities via the benefits of SMRs.

f. *Principle 6: Limitation of risk to individuals*

The sixth principle states that measures to control radiation risks must ensure that no individuals bear unacceptable risks of harm. As with the meaning of what is ‘reasonably achievable’ in the previous principle, further explanation can help articulate how to handle ‘unacceptable risks of harm’. Practically, however, the IAEA refers to a maximum threshold of radiation doses to remain within the desired level of safety. This threshold can be translated to measures to achieve the safety level, and hint on their agential aspects. An agent-centred perspective could further articulate the types of measures required to limit risks to individuals. Principle 6 can thus be formulated as: *institutional measures translated into, among other things, engineering, policy, and educational strategies* must ensure that no individuals bear unacceptable risks of harm.

In the context of SMRs, agent-centred principle 6 can be implemented through, among other things, design features such as the ability to keep radioactive material within the reactor in case of core melting, thus preventing the release of high-level radioactive material into the environment. Furthermore, scenarios such as core-melting accidents should also be addressed within local and national regulations. Other SMR-specific features, such as the ability to partially shut down reactors and the conditions to do so, should also be made clear in the regulations and communicated to the public.

g. *Principle 7: Protection of present and future generations*

The seventh safety principle states that there must be protection against radiation risk for people and the environment, in the present and in the future. In the context of NPPs, this principle is relevant for intra- and intergenerational justice issues. As for compatibility with eRA, one of the foci of the assessment is risk-exposed persons who cannot be included in the decision process. Those who are living now have no means of accessing the interests of the environment (present or future) or of those who are not born yet. Therefore, it is important to ensure a high standard of operation which is resistant to radioactive accidents or ready to respond quickly in the case of an accident. With an agent-centred perspective, this principle can be detailed as follows: *policymakers and power plant industries should minimise* radiation risk for people and the environment, present and future.

In the context of SMRs, when commercialising this new technology, particularly for new adopting countries, the government, nuclear companies, and the public need to be well-informed about the type of SMR being used and its functioning, down to the use of the nuclear fuel, coolant, and moderator. If an SMR is using molten salt, for instance, there should be detailed information about how HLW is produced and measures regulating how much is produced, and whether or not it will be recycled back into the reactor. Those involved need to know how to acquire and transport the nuclear fuel before and after the fission process inside the reactors.

h. *Principle 8: Prevention of accidents*

The eighth principle states that it is essential to employ all practical efforts to prevent and mitigate nuclear or radiation accidents. In this principle, preventive and mitigative actions are equally important, and both must be addressed. The question, then, is about the party that is primarily responsible for employing all practical efforts. While policymakers are responsible for issuing NPP licenses, practical efforts to engineer safe NPPs mainly fall to the licensee. Following this principle, eRA can further define those who should bear responsibility for preventing and mitigating nuclear accidents. An agent-centred perspective on principle 8 could thus be formulated as: all practical efforts must be made *primarily by business entities in cooperation with the government* to prevent and mitigate nuclear or radiation accidents.

Taking an example from SMRs, due to their smaller size and potential use of new materials as fuel, moderator, and coolant – such as using thorium as fuel with molten salt as moderator and coolant – nuclear companies must exercise caution in building, operating, and maintaining NPPs. This can be done, for example, by taking full responsibility for the safety of the reactor design and providing policymakers with transparent data on the safety status of the NPPs.

i. *Principle 9: Emergency preparedness and response*

The ninth principle states that arrangements must be made for emergency preparedness and response for nuclear and radiation incidents. eRA could improve this principle by specifying the parties responsible for ensuring the emergency preparedness and response of NPPs in the case of nuclear incidents. Although the public receives benefits in exchange for being exposed to radioactive risk – primarily in the form of abundant, clean, and in many cases, affordable electricity – NPP companies still enjoy the greatest benefit, particularly in monetary form. With that in mind, adding an agent-centred perspective to principle 9 can be formulated as follows: *nuclear companies bear the primary responsibility* for ensuring emergency preparedness and response for nuclear or radiation incidents.

For example, companies building, operating, and maintaining SMRs through the decades of an NPP’s lifecycle bear the primary responsibility for anticipating radiation accident scenarios, including those arising from natural disasters or geological activities. That said, the cost of nuclear accidents is too high for both the nuclear company and the insurance company to cover, so the bill falls to the state. This was the case, for instance, with the Fukushima incident, where the Japanese government stepped in to help Tokyo Electric Power Company (TEPCO), paying for compensation and the decommissioning cost (Reuters, 2011; McCurry, 2012; Jazeera, 2013). Since the state’s intervention is crucial here, the practical application of this principle includes the responsibility of nuclear companies to cooperate with the state. Maintaining cooperation with the state from the beginning could thus be interpreted as a critical element of emergency preparedness and response to potential radiation incidents.

j. *Principle 10: Protective actions to reduce existing or unregulated radiation risks*

The tenth principle states that protective actions to reduce existing or unregulated radiation risks must be justified and optimised. The scope of justified protective actions here could benefit from eRA due to its reliance on fairness in risk distribution. While the assessment is open to other criteria of fairness, it is important that individuals, particularly the most vulnerable, not be exposed to risks. With that in mind, an agent-centred principle 10 could be formulated as follows: protective actions to reduce existing or unregulated radiation risks must be optimised *without further exposing individuals, particularly the most vulnerable, to risks.*

In the context of SMRs, principle 10 can be applied, for example, in deciding where to build the NPPs. SMRs’ small size and modularity allow them to be built near inhabited areas, which makes them ideal for supplying electricity to nearby cities, to regions with intermittent power or devoid of electricity. Therefore, it is also essential to have well-planned nuclear accident scenarios for the site as well as infrastructure to support nuclear safety, such as the transportation route for fuels, reactor modules, etc.

**Table 2:** eRA-enhanced Fundamental Safety Principles for Nuclear Power Plants. The first and second columns show the IAEA nuclear principles (Fundamental Safety Principles 2006)

Fundamental Safety Principles	Definition	Agent-Centred Nuclear Safety Principles
1. Responsibility for safety	The prime responsibility for safety must rest with the person or organisation responsible for facilities and activities that give rise to radiation risks.	The prime responsibility for safety must rest with the person or organisation *that benefits from and decides to initiate* facilities and activities that give rise to radiation risks.
2. Role of government	An effective legal and governmental framework for safety, including an independent regulatory body, must be established and sustained.	There must be an effective legal and governmental framework for safety, with an independent regulatory body *assessing radioactive risk exposure, benefits derived from radioactive risk, and the distribution of both.*
3. Leadership and management for safety	Effective leadership and management for safety must be established and sustained in organisations concerned with, and facilities and activities that give rise to, radiation risks.	It is compulsory to establish and sustain leadership and management for safety in nuclear facilities that give rise to radioactive risks *by, among other things, ensuring the accessibility and distribution of knowledge about radioactive risks to all relevant agents*.
4. Justification of facilities and activities	Facilities and activities that give rise to radiation risks must yield an overall benefit.	Facilities and activities which give rise to radiation risks must yield an overall benefit *either monetarily or in-kind for the risk-exposed around the nuclear facilities.*
5. Optimisation of protection	Protection must be optimised to provide the highest level of safety that can reasonably be achieved.	*Governments and nuclear companies* must provide the highest possible level of safety *with a reasonable standard that adheres to fair radioactive risk distribution*.
6. Limitation of risks to individuals	Measures for controlling radiation risks must ensure that no individual bears an unacceptable risk of harm.	*Institutional measures translated into, among other things, engineering, policy, and educational* measures must ensure that no individuals bear unacceptable risks of harm.
7. Protection of present and future generations	People and the environment, present and future, must be protected against radiation risks.	*Policymakers and power plant industries must minimise* radiation risk for people and the environment, present and future.
8. Prevention of accidents	All practical efforts must be made to prevent and mitigate nuclear or radiation accidents.	All practical efforts must be made *primarily by business entities in cooperation with the government* to prevent and mitigate nuclear or radiation accidents.
9. Emergency preparedness and response	Arrangements must be made for emergency preparedness and response for nuclear or radiation incidents.	*Nuclear companies bear the primary responsibility for ensuring* emergency preparedness and response for nuclear or radiation incidents.
10. Protective actions to reduce existing or unregulated radiation risks	Protective actions to reduce existing or unregulated radiation risks must be justified and optimised.	Protective actions to reduce existing or unregulated radiation risks must be optimised *without further exposing individuals, particularly the most vulnerable, to risks.*

## Critique: eRA’s Agent-Based Approach Overlooks Institutional Embedding as individuals’ *Milieu*

eRA works by integrating ethical aspects to supplement quantitative, techno-economic risk assessments. Its emphasis on risk distribution among the three roles of agents – decision-makers, risk-exposed persons, and beneficiaries – shapes its agent-based approach to risk, which we use here to improve the formulation of fundamental safety principles. However, there remain some potential shortcomings of these eRA-enhanced nuclear safety principles. One particular challenge that we will address in this section is the lack of reflection on institutional embedding in the relations between agents and novel nuclear technologies, exemplified by SMRs.

Regarding SMR deployment and safety considerations, eRA makes the need to include fairness in risk distribution in nuclear safety principles more explicit by specifying the relevant agents. However, these agents and potential SMRs are not simply collections of individuals interacting in the same way across all nations. There is a set of rules, or more fundamentally, a setting, within which individuals navigate and where SMRs will be sited. This setting makes the difference in nuclear policy between, for instance, France and Germany, not only politically, but also culturally.

The problem with eRA’s agent-based approach is that its focus on individual roles, risks, benefits, and access to knowledge overlooks how individuals would likely behave in a given setting. Informing people about exposure to radioactive risks, as well as providing relevant knowledge about SMRs, presupposes a *milieu* where such interactions will take place (see Ostrom, 2011). Societal structures, flow of information, forms of governance, and education systems are part of institutional embedding. In the SMR context, due to the NPPs’ impact on their surroundings, nuclear-related issues are institutional in the sense that they are invisible (Ostrom, 2005) and correspond to the rules, norms, and strategies adopted by individuals operating within or across organisations (Ostrom, 2019, 2008; Crawford & Ostrom, 1995). This setting motivates individuals’ attitudes towards nuclear policy in a specific way.

Institutional embedding ensures that SMR developments contribute to a resilient energy system which benefits all individuals. Learning from past crisis experiences, such as the German energy system’s lack of preparedness during the Russia–Ukraine war, robust knowledge about country-specific institutional embedding would contribute to building SMRs to protect against an energy crisis. The market, financing schemes, and national or international politics are prone to change. These aspects are yet to be accounted for by eRA-enhanced nuclear safety principles.

By overlooking institutional embedding, eRA-enhanced nuclear safety principles overlook the most likely societal setting that would allow for the development and commercialisation of SMRs and other advanced reactors. These revised safety principles navigate the moral questions surrounding nuclear technological risks. But to prevent unjust distribution of risks and responsibility associated with nuclear power, it is also important to consider economic, political, and cultural aspects that support the engineering of future reactors. These considerations require analysis of institutional embedding, as this motivates certain socio-technical interactions in nuclear technology in the first place.^5^

One way to think about institutional embedding in nuclear power is by analysing how nuclear energy policy takes shape within a given political system. Such analysis considers how various organisations (government, NGO, research institutes, etc.), companies, political parties, etc., interact and thus affect an SMR development project. For example, the government of France owns the national electric utility, Électricité de France (EDF), which operates NPPs, as well as around 80% of Framatome, one of the world’s largest nuclear companies (‘Nuclear Power in France - World Nuclear Association’ 2026). Institutional embedding for nuclear power in France would differ from that in the US, where many different companies operate NPPs across 30 different states (‘Nuclear Power in the USA - World Nuclear Association’ 2026). Aspects such as the number of operators and their sizes, the way energy is governed within a state, the scope of a nuclear project, and so on, impact the analysis of the depth of institutional embedding for nuclear safety.

The topic of institutional embedding in nuclear technological systems could be pursued in further work. That said, it is worth mentioning that human interaction, knowledge distribution, and perceptions of NPPs’ risks, benefits, cost, and safety change over time and across space. eRA adds human agents into risk considerations; as a result, eRA-enhanced nuclear safety principles require further perspectives to anticipate changes in human interactions which affect nuclear technological systems.^6^ However, eRA overlooks the institutional embedding that is crucial for the deployment of SMR or any future NPP. Beyond these safety principles, societal embedding in the implementation of NPPs beyond electricity production – e.g., using SMRs for water desalination or to power AI data centres – could be a fruitful area of research.^7^

## Conclusion

Small Modular Reactors open new possibilities for NPP scenarios due to their compact size and the modularity of the components, particularly the nuclear reactors. These scenarios are not sufficiently addressed by existing fundamental safety principles. Many philosophical accounts that analyse the ethical aspects of NPPs are more focused on cases related to large reactors, and less on scenarios raised by new reactor designs such as SMRs. At the same time, the IAEA’s overarching fundamental safety principles, which were issued nearly 10 years ago, are too general for usage in new reactor designs exemplified by SMRs and thus need to be further specified.

An agent-based ethical approach to nuclear safety principles, which tackles the question of ‘who is exposed to what risk’, enhances the precision of existing safety principles such that they can apply to new reactor designs such as SMRs. This approach spells out the agent-centred perspective by adopting Hermansson and Hansson’s ethical Risk Assessment (eRA) and applying it to existing fundamental safety principles, exemplified using the case of SMRs. With this approach, we propose agent-centred fundamental safety principles.

That said, agent-centred fundamental safety principles still do not account for institutional embedding, which enables NPPs’ development across various countries. Institutional embedding shapes technological systems in changing societal landscapes. Future analysis of institutional embedding is therefore necessary to more thoroughly conceptualise what it means to build a nuclear energy system that not only promotes safety but also anticipates changes in nuclear technological landscapes.

## Data Availability

The figure in this work is also available in supplementary materials.

## Footnotes

1 These principles are: responsibility for safety, role of government, leadership and management for safety, justification of facilities and activities, optimisation of protection, limitation of risks to individuals, protection of present and future generations, prevention of accidents, emergency preparedness and response, and protective actions to reduce existing or unregulated radiation risks (Fundamental Safety Principles, 2006).

2 For example, Kermisch and Taebi (2017) state that different NPP designs could lead to different risk profiles, while Taebi and Mayer (2017) underline the importance of a comprehensive institutional approach to safety, otherwise known as a designed approach. Taebi (2012), Richter (2017), Shrader-Frechette (2005), and Tondel and Lindahl (2019) highlight the importance of a global institutional approach to addressing the existing radioactive risks of HLW with unknown societal and environmental effects for future generations. Shrader-Frechette treats the topic of nuclear waste as a vulnerability issue (Shrader-Frechette, 2005) that utilitarian accounts cannot solve, since in terms of numbers, future generations make up the majority of the population affected by nuclear waste disposal (Shrader-Frechette, 1994). Thus it is important that sustainable practices for waste management be motivated by the responsibilities of policymakers and engineers to minimise undue burdens imposed on future generations by HLW (Taebi et al., 2012). Further ethical discussion on nuclear waste also addresses retrievability and reversibility of nuclear waste with equity as the key moral stance (Kermisch et al., 2016). Topics adjacent to nuclear waste often use intergenerational justice as a consideration for an open or closed nuclear fuel cycle (Van Est et al., 2023; Taebi & Kloosterman, 2008).

3 Hansson does not seem to propose a particular idea of how distributive justice in assessing technology should look (see Hansson, 2017b).

4 Note that the IAEA’s wording of an “overall benefit” in relation to radiation risks also has ethical underpinnings. Radiological protection, and by extension, radioactive risk management in NPPs, is a matter of ethical principles such as beneficence/non-maleficence, justice, prudence, and human dignity. Cho et al. (2018) from the International Commission on Radiological Protection (ICRP) use the term “ethical values” for these principles. Meanwhile, Hansson also makes a connection between the moral examination of one’s actions and utilitarian, deontic, and virtue ethics: weighing is associated with utilitarianism, limiting with deontological ethics, and orienting with virtue ethics (Hansson, 2007). Therefore, despite the radioactive risks, one could also ethically analyse the overall benefit of NPPs from the perspective of values and principles, utility, duties, and virtues.

5 Authors who take a phenomenological perspective, like Bontems, for instance, include changing societal context in the formation of technologies. Bontems uses Simondon’s idea of viewing technology, including nuclear technologies, as both supported by and supporting science, aesthetics, and culture (Bontems, 2018). This type of approach links engineered characteristics in technologies to individual faculties of human beings as the locus of technological innovation (Petit & Guillaume, 2018﻿; Simondon, 2007), thus adjusting engineered features to suit human faculties. One could also go deeper into ontology by connecting the idea of societal embedding as the ‘flesh’ of nuclear energy systems. This idea is derived from Merleau-Ponty’s concept of ‘flesh’ as the stage where phenomena take place, i.e., surfacing from invisible to visible (Merleau-Ponty, 1964).

6 Hermansson and Hansson seem to take account of the importance of identifying ignorance while assessing risk in their own ethical risk assessment. They seem to conceptualise ignorance interchangeably with great uncertainty, referring to both as events with unknown probability and consequences (Hansson, 2013). This conceptualisation appears to be in line with other accounts such as that of Riesch (Riesch, 2012).

7 For some authors, such as Doorn and Hansson, this type of nuclear engineering is regarded as safety engineering (Doorn & Hansson, 2015), which considers continuous development during the early stage of the design. Others such as Hillerbrand and Peterson integrate human capability and vulnerability into considerations of engineering risk (Hillerbrand & Peterson, 2014).
